# Cerebral oximetry monitoring by means of INVOS-4100 as a predictor of ischemic events during carotid endarterectomy

**DOI:** 10.3389/fsurg.2023.1170019

**Published:** 2023-04-11

**Authors:** Maria Francesca Russo, Patrizia Gentile, Marco Fenga, Silvia Izzo, Flavia Denaro, Klaudia Luka, Flaminia Frattaroli, Alessandro Costanzo, Lidia Castagneto-Gissey, Bruno Salvati

**Affiliations:** ^1^ Department of Surgery, Sapienza University of Rome, Rome, Italy; ^2^ Department of Anesthesia and Intensive Care Medicine, Sapienza University of Rome, Rome, Italy; ^3^ Centre de Chirurgie Vasculaire et Endovasculaire, Groupe Hopitalier Paris, Saint Joseph, Paris, France

**Keywords:** carotid endarterectomy (CEA), cerebral oximetry monitoring, stroke, vascular surgery, oximetry

## Abstract

**Background:**

Several methods have been proposed to monitor cerebral perfusion during carotid endarterectomy (CEA), with the purpose of minimizing the risk of perioperative stroke. The INVOS-4100 is able to detect cerebral oxygen saturation providing an intraoperative real-time monitoring system of cerebral oximetry. The aim of this study was to evaluate the performance of the INVOS-4100 in predicting cerebral ischemia during CEA.

**Methods:**

Between January 2020 and May 2022, 68 consecutive patients were scheduled for CEA either under general anesthesia or regional anesthesia with deep and superficial cervical block. Vascular oxygen saturation was recorded continually through INVOS before and during clamping of the ICA. Awake testing was performed in the group of patients undergoing CEA under regional anesthesia.

**Results:**

Sixty-eight patients were included; 43 were males (63.2%). Severe stenosis of the artery was present in 92%. Forty-one (60.3%) patients were monitored by INVOS, while 22 (39.7%) underwent awake testing. Mean clamping time was 20 ± 6.6 min. Patients undergoing awake testing had a lower hospital stay and ICU stay during admission (*p* = 0.011 and *p* = 0.007 respectively). Comorbidities correlated with a higher ICU stay (*p* < 0.05). The INVOS monitoring was able to predict ischemic events with a sensitivity of 98% (AUC = 0.976).

**Conclusions:**

The present study demonstrates that cerebral oximetry monitoring was a strong predictor of cerebral ischemia, although it was not possible to determine the non-inferiority of cerebral oximetry compared to awake testing. Nonetheless, the use of cerebral oximetry evaluates only perfusion in the superficial brain tissue and an absolute rSO2 value corresponding to significant cerebral ischemia has not been established. Therefore, larger prospective studies that correlate cerebral oximetry with neurologic outcomes are needed.

## 1. Introduction

Carotid endarterectomy (CEA) as a treatment for high-grade carotid artery disease is nowadays an established treatment (1, 2). In an estimated 2%–3% of patients undergoing CEA perioperative stroke may occur due to either cerebral ischemia or embolism during surgery (3–5). In order to minimize the risk of a perioperative stroke, several methods are available that help monitor cerebral perfusion. Indeed, somatosensory evoked potentials (SEV), the use of electroencephalography, transcranial Doppler sonography, neurologic assessment in awake patients with regional block are established techniques (6). However, none of these can predict the occurrence of perioperative ischemia (7). Cerebral oximetry is a simple, non-invasive, real-time monitoring of cerebral oxygen saturation (rSO2) during extra-cranial carotid surgery (8), based on the principles of near-infrared spectroscopy, first described by Jobsis (9). The most widely used device, the INVOS-4100 (Somanetics, Inc, Troy, MI), is able to detect cerebral oxygen saturation at a depth of 11–14 mm below the surface of the brain (10, 11).

The aim of this study was to evaluate the performance of the INVOS 4100 in predicting cerebral ischemia during carotid endarterectomy.

## 2. Materials and methods

### 2.1. Study design

Between January 2020 and May 2022, 68 consecutive patients were scheduled for CEA either under general anesthesia or regional anesthesia with deep and superficial cervical block. Inclusion criteria were primary CEA in patients with symptomatic or asymptomatic internal carotid artery stenosis. Patients who had an internal carotid artery with a diameter ≤5 mm and who received an internal carotid artery patch angioplasty were excluded from this study due to the greater risk of restenosis.

A temporary reduction in our annual surgical volume was recorded in the study period due to COVID-19 pandemic (12). Written approval was obtained from the local hospital Research and Ethics Committee. Additional informed consent was obtained before all surgical procedures.

### 2.2. Carotid endarterectomy surgical technique

The patient is placed in a supine position with a hyperextension of the neck with a head rotation opposite to the operation side. A longitudinal skin incision is made along the anterior aspect of the sternocleidomastoid muscle. The carotid sheath is reached by cutting through the subcutaneous fat tissue and the platysma muscle. The carotid arteries are exposed. The common carotid artery is clamped below the bifurcation, then the external carotid is clamped, and finally the internal carotid is clamped distally. The surgeon opens the length of the internal carotid artery (ICA), to locate both ends of the plaque. The plaque is then divided and removed. The artery is then closed through a direct primary suture using a 4-0 or 5-0 prolene.

The indication for surgery were clinically asymptomatic severe or pre-occlusive carotid artery stenosis ≥70%, clinically asymptomatic stenosis with ipsilateral signs of cerebral ischemia on CT scan and clinically symptomatic stenosis.

The circle of Willis and vertebral arteries were evaluated in all patients preoperatively by means of a CT-angiography. In the present study group, none of the patients had an in-complete or defective circle of Willis. Collateral flow to the cerebral hemispheres was pre-sent in all cases. Vertebral arteries were patent and viable in all subjects included in this study.

### 2.3. NIRS-INVOS 4100

The cerebral oximeter INVOS-4100 was used to measure rSO2 throughout the procedure. After cleaning the skin, bilateral rSO2 oximeter electrodes were placed as high up on the forehead as possible. After a stable rSO2 reading was achieved, the sensors were tightened with tape. Vascular oxygen saturation was then recorded continually before clamping and during clamping of the ICA. All patients were extubated in the operating room and tested for any new neurological deficit.

### 2.4. Awake testing

The awake testing procedure can only be performed under regional anesthesia. The procedure was explained to patients the day before surgery. The anesthesiologist asked the patient to open his/her eyes and to repeat this task every 30 s until the patient responded to the command and pupillary light reflexes were evaluated. Next, the patient was asked to squeeze both hands. Only after normal neurologic status was confirmed, the surgeon proceeded with carotid endarterectomy. Once surgery is completed, patients are tested for possible neurological deficits.

### 2.5. Statistical analysis

Continuous variables are expressed as means ± SD in case of normal distributions, otherwise as medians. Normality was assessed using visual inspection of density plot and Q-Q plot along with the Shapiro-Wilk test. Since most variables were not normally distributed, for quantitative variables possible differences between INVOS and clinical monitoring were assessed by means of a Mann–Whitney *U* Test. Association between the type of methods used and categorical variables was tested with a *χ*^2^ test. A *p*-value <0.05 was considered statistically significant.

All data manipulation and analyses were conducted using SPSS version 27 (13).

### 2.6. Receiver operating characteristic curve analysis

Receiver operating characteristic (ROC) curve analysis was generated by the Neural Net-work Analysis as a predictive data mining application. A ROC curve analysis was used to assess how accurate a marker is capable of describing the occurrence of ischemic events between 2 states: “Monitored by INVOS” and “Not monitored by INVOS” (14). In a ROC curve, the true positive rate (sensitivity) is plotted as a function of the false-positive rate (1-specificity) for different cutoff points of a parameter. Among the indices of accuracy proposed to summarize ROC curves is the AUC, a unidimensional index. The maximum AUC = 1 means that the diagnostic test is perfect in the description of ischemic events (15).

## 3. Results

### 3.1. Operative outcomes

Baseline characteristics and demographics are reported in Table 1. Sixty-eight patients were included in the study. Forty-three patients were males (63.2%).

**Table 1:** Baseline characteristics in INVOS monitoring versus awake testing groups.

	INVOS monitoring (*N* = 41)	Awake testing (*N* = 27)	*p*-value
Males, *n* (%)	29 (70.7)	11 (40.7)	
Age, year, mean ± SD	73 ± 10	73 ± 9	0.804
Hypertension, *n* (%)	29 (70.7)	13 (48.1)	**0** **.** **015**
Diabetes, *n* (%)	9 (22)	8 (29.6)	0.747
COPD, *n* (%)	4 (9.7)	5 (18.5)	0.492
**Smoking status, *n* (%)**			
Current Smoker	8 (19)	4 (14.8)	0.714
Former Smoker	10 (24)	3 (11.1)	
Never smoked	23 (56)	15 (55.5)	
**Contralateral stenosis, *n* (%)**			
None	9 (22)	3 (11.1)	0.353
Mild	16 (39)	7 (25.9)	
Moderate	7 (17.1)	6 (22.2)	
Severe	7 (17.1)	4 (14.8)	

COPD, chronic obstructive pulmonary disease; N/A, not applicable; SD, standard deviation.

The bold values indicate significant differences between analyzed values.

Thirty-three patients (48%) were diagnosed with stenosis of the left carotid artery, while 35 (51%) had stenosis of the right carotid artery. Forty-nine patients (72%) were asymptomatic.

Almost all patients, 92%, had severe stenosis of the artery involved. Twenty-four patients (35%) had mild contralateral stenosis, fifteen patients (22%) had moderate contralateral stenosis, twelve patients (17.6%) had severe contralateral stenosis. When analyzing the structure of the plaque, 20 cases were fibro-calcific (29.4%).

Only 6 patients (8.8%) experienced postoperative ischemic events. Mortality was 1.4% (*n* = 1). The cause of death was acute myocardial infarction in 1 patient. Of the 6 patients who developed a postoperative ischemic event, 4 had embolization highlighted by post-operative CT and/or MRI which resulted in a non-disabling minor stroke or transient ischemic attack; hyperperfusion injury syndrome was present in 1 patient; and no apparent attributable cause was found in 1 patient. There was no statistical difference in the rate of ischemic events between symptomatic and asymptomatic patients (*p* = 0.4413).

General anesthesia was performed in 41 (60.3%) who were monitored by INVOS, while 27 (39.7%) patients underwent regional anesthesia with awake testing. Data were collected before and during clamping. Mean clamping time was 20 ± 6.6 min.

Table 2 reports data comparing patients who underwent INVOS vs. awake testing. Patients undergoing awake testing had a lower hospital stay and ICU stay during admission (*p* = 0.011 and *p* = 0.007 respectively). Furthermore, comorbidities correlated with a higher ICU stay (*p* < 0.05).

**Table 2:** Postoperative outcomes in INVOS monitoring versus awake testing groups.

	INVOS monitoring (*N* = 41)	Awake testing (*N* = 27)	*p*–value
Postoperative ischemic events, *n* (%)	4 (9.7)	2 (7.4)	0.932
LOS, days, mean ± SD	8 ± 4	5 ± 2	**0** **.** **011**
ICU, days, mean ± SD	0.9 ± 1	0.3 ± 0.5	**0**.**007**
**Histological plaque features, *n* (%)**			
Calcific	9 (22)	6 (22.2)	0.296
Fibrocalcific	14 (34.1)	5 (18.5)	
Ulcerated	0	0	
Mixed	4 (9.8)	9 (33.3)	
N/A	2 (4.8)	0	
**Ultrasonographic plaque features, *n* (%)**			
Hyperechogenic	4 (9.8)	1 (3.7)	0.327
Hypoechogenic	1 (2.4)	0	
Isoechogenic	7 (17.1)	1 (3.7)	
Clamping time, (min) mean ± SD	20 ± 5	21 ± 8	0.719
**Cerebral oximetry before clamping, (%)**			
Left	66 ± 7.8	N/A	
Right	67 ± 7		
**Cerebral oximetry during clamping, (%)**			
Left	62 ± 10	N/A	
Right	63 ± 8.8		

LOS, length of stay; ICU, intensive care unit; N/A, not applicable; SD, standard deviation.

The bold values indicate significant differences between analyzed values.

### 3.2. INVOS as a determinant of ischemic events

**Figure 1:**
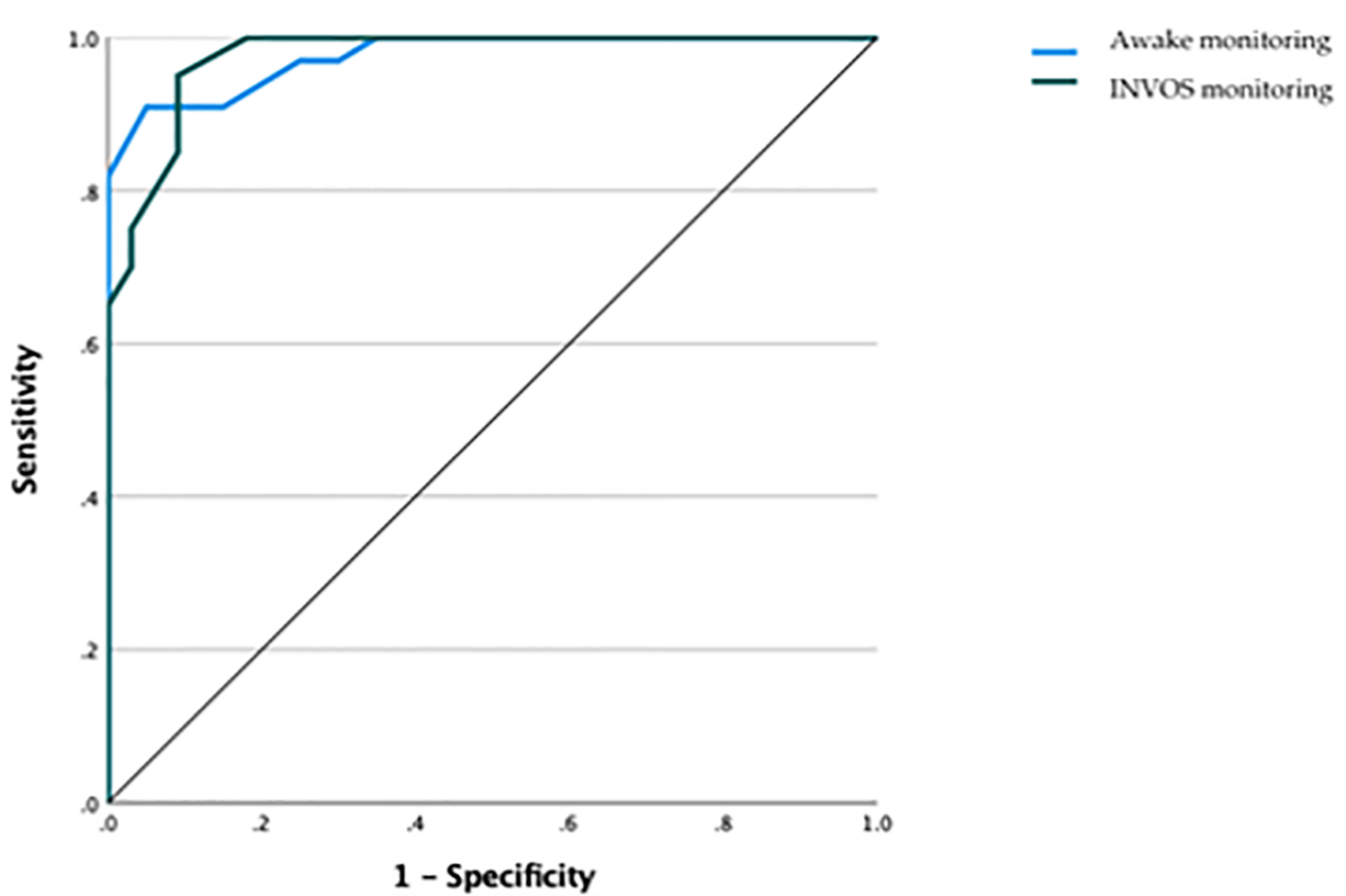
Receiver operating characteristic (ROC) curve highlighting how cerebral oximetry was able to predict ischemic events with a sensitivity of almost 98% (AUC = 0.976).

We used artificial neural network analysis to assess if INVOS could predict ischemic events. The INVOS monitoring was able to predict ischemic events in our cohort of patients with a sensitivity of almost 97.6% (AUC = 0.976) (Figure 1).

## 4. Discussion

This study was designed to evaluate INVOS 4100-cerebral oximetry performance in the prediction of ischemic events during CEA surgical procedures. Patients monitored with INVOS had a higher length of ICU and hospital stay. A lower in-hospital stay in the awake group could be perhaps attributable to regional anesthesia which concurs to an enhanced recovery to physiological activities, hence leading to a shorter overall length of stay and a lower rate of overall perioperative complications.

It was recommended by the INVOS manufacturer that a cerebral oximetry level of either <50% or a 20% decline from baseline values should be used as a sign of ongoing neuro-logical decline. However, some authors accepted a 5% decline from baseline as a significant sign of neurological cognitive deterioration (7). In our study, as per manufacturers' suggestion, a prolonged cerebral desaturation below 50% or a drop of more than 20% from baseline, was considered as an absolute value that could trigger the need for shunt placement. Nevertheless, in our experience, cerebral oximetry during clamping was 62 ± 10–63 ± 8.8% on the left and right hemispheres respectively, with no case dropping below the indicated threshold.

Despite no substantial drop in intraoperative cerebral oximetry in the INVOS group, postoperative ischemic events in this group were 9.7% (*n* = 4) vs. 7.4% (*n* = 2) in the awake testing group (*p* = 0.932). The 6 patients who displayed a postoperative ischemic event developed a non-disabling minor stroke or transient ischemic attack. The overall rate in our cohort of patients was 8.8% which is consistent with the incidence of ischemic events of 10.2% following CEA reported in the literature (16, 17).

Interestingly, monitoring patients with cerebral oximetry was able to predict ischemic events with a sensitivity of almost 98% (Figure 1). Such a high predictive value could contribute to a prompt diagnosis and appropriate management of intraoperative ischemic events. In fact, controversies on the use of systematic intraluminal shunting during CEA still exist, as it can complicate the procedure and raises the possibility of stroke, yet it helps to reduce cerebral hypoperfusion during carotid cross-clamping (7, 18). In this regard, cerebral monitoring could represent a crucial tool in leading to the decision of performing a selective intraluminal shunt in order to prevent intraluminal shunt-related stroke in those patients with major risk factors.

Our results, however, are in contrast with data emerging from the only two articles retrieved from the literature assessing cerebral ischemia through the use of the INVOS system. Both studies found cerebral oximetry had low sensitivity and specificity and a high negative predictive value for cerebral ischemic events. Specifically, Stilo et al. (7) demonstrated that cerebral oximetry was able to detect ischemic events with a sensitivity of 60%. Also, Samra et al. showed that monitoring rSO2 to detect cerebral ischemia during carotid endarterectomy had a strong negative predictive value but a low positive predictive role (19). Such a different result with regard to predictive values could be explained by the improved version of INVOS which was used in our cohort of patients compared to the aforementioned authors. Perhaps the advancement of technologies could aid in registering cerebral oximetry with greater precision, translating into a raised predictive value.

According to a recent systematic review and meta-analysis evaluating the diagnostic accuracy of intraoperative cerebral oximetry monitoring during CEA under regional anesthesia by means of different types of available near-infrared spectroscopy devices, the point estimate of the sensitivity of cerebral oximetry for predicting postoperative stroke was low (41%) with a broad confidence interval. The poor positive predictive value and high negative predictive value of intraoperative near-infrared spectroscopy in postoperative stroke prediction are also explained by the low frequency of stroke episodes. Additionally, the near-infrared spectroscopy value that could be used to forecast postoperative cerebral ischemia could not be found by the authors. As there was no threshold effect in the range of cutoff values reported in the primary trials, the ideal cutoff that would benefit from the use of shunting could not be identified (20).

Pennekamp et al. reported that a 16% decrease in near-infrared spectroscopy had a positive predictive value of 76% and a negative predictive value of 99% for the detection of cerebral hypoperfusion in a prospective cohort study involving patients submitted to CEA under general anesthesia, suggesting that this method might be utilized to direct the use of selective shunts (21).

The criteria for shunt insertion reported in the literature are always based on neurological examination findings of evidence of cerebral ischemia or near-infrared spectroscopy data, and all investigations documented the use of selective carotid shunting (22–24). The number of patients experiencing a hemodynamic stroke during carotid cross-clamping, which may be prevented by the use of shunts, represents a significant issue, making this area of research of the utmost relevance. Therefore, it is necessary to develop an effective brain monitoring protocol to recognize these patients.

A comparison of cerebral oximetry evaluation with other accepted techniques of cerebral ischemia monitoring is difficult to achieve. The awake testing is considered the gold standard technique in the assessment of ischemia bearing the highest sensitivity and specificity (25). However, there is no consensus on the perfect monitoring of cerebral ischemia in patients undergoing carotid endarterectomy under general anesthesia. Cerebral oximetry is an easy, non-invasive technique of continuously monitoring cerebral oxygen saturation. Several studies correlated CO monitoring to other methods of cerebral perfusion assessment during carotid endarterectomy. Cho et al. (26) showed that a critical drop of −10 rSO2 units or a decrease in rSO2 below 50% were suggestive of cerebral ischemia based on changes in SSEPs. Williams and colleagues compared ipsilateral TCD monitoring in 54 patients undergoing carotid endarterectomy. Data showed a significant association between changes in ipsilateral cerebral oxygen saturation and the percent change in middle cerebral artery velocity. Authors concluded that cerebral oximetry could be more efficient since it can be used in all patients and does not require any particular assistance (27).

Some study limitations must be acknowledged. The use of two different types of anesthesia (general vs. regional) might influence the comparability of the two groups. Specifically, general anesthesia has been shown to reduce cerebral oxygenation during general anesthesia. However, INVOS oxygenation recordings were done throughout the whole procedure starting with a baseline value at the beginning of general anesthesia induction and all comparisons were based on this initial value.

## 5. Conclusions

Although the outcomes emerging from the present study demonstrate that cerebral oximetry monitoring was a strong predictor of cerebral ischemia, it was not possible to determine the non-inferiority of cerebral oximetry with respect to awake testing, which is still considered at present the gold standard. Nonetheless, the use of cerebral oximetry evaluates only perfusion in the superficial brain tissue and an absolute rSO2 value corresponding to significant cerebral ischemia has not been established yet. Therefore, larger prospective studies that correlate cerebral oximetry with neurologic outcomes are warranted.

## Data Availability

The original contributions presented in the study are included in the article/Supplementary Material, further inquiries can be directed to the corresponding author/s.
